# Functionalized Magnesium Phosphate Cement Induces In Situ Vascularized Bone Regeneration via Surface Lyophilization of Chondroitin Sulfate

**DOI:** 10.3390/biomedicines12010074

**Published:** 2023-12-28

**Authors:** Changtian Gong, Jian Yang, Xiping Zhang, Zhun Wei, Xingyu Wang, Xinghan Huang, Ling Yu, Weichun Guo

**Affiliations:** 1 Department of Orthopedics, Renmin Hospital of Wuhan University, Wuhan 430060, China; gongct@whu.edu.cn (C.G.); yangjian1986@whu.edu.cn (J.Y.); 2016302180026@whu.edu.cn (X.Z.); weizhun1998@whu.edu.cn (Z.W.); 2017302180019@whu.edu.cn (X.W.); 2017302180015@whu.edu.cn (X.H.); scaling@126.com (L.Y.); 2 Center of Regenerative Medicine, Renmin Hospital of Wuhan University, Wuhan 430060, China

**Keywords:** magnesium phosphate cement, k-struvite, chondroitin sulfate, osteogenesis, angiogenesis

## Abstract

Bone defect repair poses significant challenges in orthopedics, thereby increasing the demand for bone substitutes. Magnesium phosphate cements (MPCs) are widely used for bone defect repair because of their excellent mechanical properties and biodegradability. However, high crystallinity and uncontrolled magnesium ion (Mg^2+^) release limit the surface bioactivity of MPCs in bone regeneration. Here, we fabricate chondroitin sulfate (CS) as a surface coating via the lyophilization method, namely CMPC. We find that the CS coating is uniformly distributed and improves the mechanical properties of MPC through anionic electrostatic adsorption, while mediating degradation-related controlled ion release of Mg^2+^. Using a combination of in vitro and in vivo analyses, we show that the CS coating maintained cytocompatibility while increasing the cell adhesion area of MC3T3-E1s. Furthermore, we display accelerated osteogenesis and angiogenesis of CMPC, which are related to appropriate ion concentration of Mg^2+^. Our findings reveal that the preparation of a lyophilized CS coating is an effective method to promote surface bioactivity and mediate Mg^2+^ concentration dependent osteogenesis and angiogenesis, which have great potential in bone regeneration.

## 1. Introduction

The treatment of critical-sized defects remains a common clinical challenge [1]. Currently, autologous bone grafts and allografts are still the preferred choice. While these treatments require secondary surgery with an increased risk of infection. The use of bone cements obviates the necessity of excising osseous tissue from a secondary site, thereby eliminating the probability of disease transmission [2,3]. To date, poly (methyl methacrylate) (PMMA) is the most commonly used bone cement owing to its exceptional mechanical properties. However, monomer toxicity and biological inertness of PMMA lead to osteoporosis-related complications, which impede its use for curing of critically sized defects [4,5].

Magnesium phosphate cements (MPCs) have recently attracted considerable attention owing to the ideal combination of early high strength and excellent biocompatibility [6]. Nevertheless, the application of traditional MPCs is usually limited by their mismatched balance between degradation and the new bone regeneration [7]. To solve this problem, the most interesting development at present is changing the phase ratio or the type of MPC, leading to enhanced bone regeneration [8,9]. For example, Trimagnesium phosphate (Mg_3_(PO_4_)_2_, TMP) has been used to replace MgO to develop a trimagnesium phosphate-potassium dihydrogen phosphate (KDP)-H_2_O (TMP-KDP-H_2_O) ternary cement system (TMPC), that exhibited higher biodegradability and osteogenic capability than conventional k-struvite [10]. However, considering the hierarchical structure between organic and inorganic phases and the physiological process after implantation, a strategy that alters the inorganic composition to improve physical dissolution has limited effectiveness. The surface bioactivity of MPCs, that promotes multiple cellular components to participate in enzymatic hydrolysis, should also be considered.

The incorporation of natural polymers into MPCs has been demonstrated to be an effective approach for enhancing surface bioactivity, owing to their similarity to the extracellular matrix (ECM) [11,12]. However, the conventional approach of adding polymers through the solid phase not only impedes the hydration reaction of MPCs, but also reduces the relative surface content, which is not fully conducive to biological activity [13]. Chondroitin sulfate (CS) is widely recognized as the most physiologically important glycosaminoglycans (GAGs) in ECM, which has been used for the treatment of osteoarthritis in cartilage tissue engineering, and is proven to be involved in the signaling functions of various growth factors and chemokines [14,15].

Therefore, our design aims to find a simple and efficient method to improve the surface bioactivity of k-struvite. In this study, we freeze-dried the CS solution to form a coating on the surface of the k-struvite to prepare a CMPC. Furthermore, we comprehensively studied the physicochemical properties, degradability, biocompatibility, and in vitro/vivo osteogenic and angiogenic abilities of the CMPC. Specially, MC3T3-E1s and HUVECs were set as cell models to evaluate osteogenesis and angiogenesis in vitro, owing to the fact that osteoblast and endothelial cells are essential cells for blood vessel formation and new bone ingrowth. Herein, we propose the CMPC as an innovative bioactive cement with good surface bioactivity and biodegradability for treating critical-sized defects.

## 2. Materials and Methods

### 2.1. Materials and Powder Preparation

The chondroitin sulfate (CS, CAS: 9007-28-7, MW: 463.36, Purity: 96.5–100%) was procured from Shanghai Macklin Biochemical Technology Co., Ltd, Shanghai, China., and the magnesium oxide (MgO) and potassium dihydrogen phosphate (KH_2_PO_4_) were purchased from Sinopharm Group Co., Ltd., Wuhan, China. All the reagents were of analytical grade. The MgO was sintered at 1600 °C for 8 h, followed by crushing via ball milling for 6 h (300 rpm/mn). Similarly, the KH_2_PO_4_ was crushed via ball milling for 6 h (300 rpm/min). The powder phase was prepared by mixing MgO and KH_2_PO_4_ at a molar ratio of 1.5:1.

### 2.2. Scaffold Preparation

The MPC scaffolds were fabricated by combining the powder phase with deionized water in a powder-to-liquid ratio (PLR) of 1.5 g/mL. The mixture was transferred to different types of Teflon molds (size A: d = 6 mm, h = 12 mm; size B: d = 6 mm, h = 2 mm) for self-setting. Following demolding, the scaffolds were treated with standard curing procedure (37 °C and 100% humidity for 48 h) for further processing. CS powder (1 g) was added to deionized water (10 mL) to prepare a CS solution (10%, *m/v*). Following thorough stirring, MPC scaffolds were immersed in different volumes of the CS solution at ratios of 6 mL/size A to 1 mL/size B to maintain the same thickness of the CS coating. Subsequently, the mixture was lyophilized (FA-1D-50; Hefan Instruments, Wuhan, China) for 6 h to obtain the CMPC.

### 2.3. Scaffold Characterization

Scanning electron microscopy (SEM) investigations were performed using a field-emission scanning electron microscope (FESEM, Zeiss SIGMA, London, UK) operating at an acceleration voltage of 25 kV after being coated with a layer of gold. X-ray diffractions (XRD) were measured using a Panalytical X-Pert PRO Diffractometer (Malvern, London, UK) with Cu–Kα radiation, followed by matching with PDF cards. Fourier-transform infrared spectroscopy (FTIR) analysis was conducted using a NICOLET 5700 FTIR Spectrometer equipped with an attenuated total reflection (ATR) device (Tokyo, Japan).

### 2.4. Mechanical Evaluation

According to ISO13779-1, the compressive strengths of the scaffolds (d = 6 mm, h = 12 mm) were tested using a universal material testing machine (RGM4100, REGER Industrial Systems, Shenzhen, China). The testing was conducted under dry conditions, with a loading rate of 2 mm/min, and the resulting stress–displacement curves were recorded. The average value for each group was determined using five samples.

### 2.5. In Vitro Degradation and Ion Release

The scaffolds (diameter = 6 mm, height = 2 mm) and CS coating (5 × 5 cm, 200 mg) were subjected to vacuum drying, and the initial mass was recorded as M0. Subsequently, the scaffolds were immersed in 0.05 M Tris-HCL buffer (pH = 7.4), and the CS coatings were, respectively, immersed in a 0.1 M NH_4_HCO_3_ buffer (pH = 7.4) with or without lipase (1.25 mg/mL). The proportions used in the above experiments was 200 mg/mL. The solution was refreshed every 2 days, and the scaffolds and CS coating were weighed and recorded as Mt after washing with PBS and vacuum-drying at the designated time point (3rd, 7th, 14th, and 21st days). The weight loss ratio is calculated as follows:

$$Weight\;Loss\left(\%\right)=\frac{\left(M_{0}-M_{t}\right)}{M_{0}}\times 100$$

The ion concentrations of Mg^2+^ and PO_4_^3−^ were measured simultaneously at the specified time points (3rd, 7th, 14th, and 21st days) using an inductively coupled plasma optical emission spectrometer (ICP-OES, Agilent 5110, Santa Clara, CA, USA).

### 2.6. Leaching Liquids Preparation

The leaching liquids for the scaffolds were prepared according to ISO10993–12. Briefly, sterile scaffolds were immersed in a complete culture medium at a concentration of 200 mg/mL for 72 h. Following centrifugation (1200 rpm/min), the supernatant solution was collected and stored at 4 °C.

### 2.7. In Vitro Evaluation of the Scaffolds

#### 2.7.1. Cell Culture

MC3T3-E1s and HUVECs were purchased from Procell Biotech and cultured in medium (CM-0378 and CM-0122, Procell, Wuhan, China). Both mediums were supplemented with 10% (*v*/*v*) fetal bovine serum (FBS; Gibco, Logan, UT, USA) and 1% (*v*/*v*) penicillin-streptomycin (P/S; Gibco). At 80–90% confluence, the cells were treated with trypsin (Gibco, 5 min) for expansion and cultured at 37 °C in a humidified atmosphere with 5% CO_2_.

#### 2.7.2. Cytocompatibility

FESEM was performed to visualize the cell morphology. MC3T3-E1s were seeded on the scaffold surface in a 24-well plate (8 × 10^4^ cells and 2 mL/well) for 5 days. Subsequently, the cell seeded scaffolds were fixed in 4% paraformaldehyde for 30 min, and sequentially dehydrated in ethanol solutions of 80%, 90%, 95%, and 100% for 10 min. Next, the cell-seeded scaffolds were placed into tertbutanol for 30 min (Macklin, Wuhan, China) at 4 °C followed by lyophilization for 6 h. Finally, the gold layer was sputtered to examine cell morphology under an acceleration voltage of 10 kV.

The viability of MC3T3-E1s was evaluated using live/dead staining (Invitrogen, Eugene, CA, USA). After being cultured with leaching liquids in a 48-well plate (MC3T3-E1s, 4 × 10^4^ cells and 2 mL/well) for 5 days, 500 μL of the live/dead reagent was added to each well. An inverted fluorescence microscope (Olympus IX71, Tokyo, Japan) was used to examine the live and dead cells. The adhesion area was quantified using ImageJ software 1.8.0 (NIH, Bethesda, MD, USA) through calculation (percentage of fluorescence area to total image area). Three random areas were used to obtain an average.

A Cell Counting Kit-8 (CCK-8) was used to assess cell proliferation. Briefly, MC3T3-E1s were seeded in 96-well plates at a density of 3 × 10^3^ cells/well, and the culture medium was replaced with a leaching liquid after 12 h. Subsequently, on the 1st, 3rd, and 5th days of the culture, the leaching liquid was replaced with 100 μL of α-MEM containing 10% CCK-8 solution. The cells were then incubated at 37 °C for 2 h, and the absorbance values were measured using a microplate reader (EnSight, PerkinElmer, Valencia, CA, USA) at 450 nm. MC3T3-E1s cultured in medium (CM-0378) were used as controls. The experiment was conducted in six replicates for each group. The formula is as follows:

$$Cell\;viability\;\left(\%\right)=\frac{OD\;value\;\left(MPC\;or\;CMPC\;group\right)}{OD\;value\;\left(control\;group\right)}\times 100$$

#### 2.7.3. Osteogenic Differentiation

The MC3T3-E1s were cultured in 6-well plates with medium (CM-0378) at 4 × 10^4^ cells/well. The osteogenic induction medium (OIM) was composed of 0.05 mM vitamin C, 10 mM β-glycerophosphate, and 1 × 10^8^ M dexamethasones (all from Sigma, San Francisco, CA, USA). When the cell density reached 60–75% approximately, the medium was switched to conditioned OIM (1:1 to leaching liquids, *v*/*v*), and MC3T3-E1s were induced to differentiate for 7/14 days. The conditioned OIM was replaced every 2 days. Osteogenic differentiation of MC3T3-E1s was evaluated by ALP staining according to the manufacturer’s instructions, and observed under a light microscope (Olympus, IX71, Osaka, Japan).

ALP activity was assessed using an ALP assay kit (Beyotime, Wuhan, China). ALP activity results were standardized to total cell protein, and media (normal OIM) were used as control. A number of 3 × 10^3^ cells per well of the MC3T3-E1s were cultured in 96-well plate with 100 μL of conditioned OIM. After 7 days of culture, cell monolayers were washed twice with PBS and were processed with 10% neutrophilic formalin (1 mL per well) for 15 min. Then, the p-nitrophenyl phosphate (pNPP) solution mixed with extraction solution and ALP buffer solution was added and reacted for 1 h. The absorbance was measured at 405 nm and three replicates for each group.

MC3T3-E1 was cultured in 6-well plates (4 × 10^4^ cells per well) with the conditioned OIM. After 14 days of coculture, alizarin red S staining reagent (1 mL per well) was added and followed by incubating in the dark for 30 min. The deposition of calcium nodules in each group was observed under an inverted microscope (Olympus, IX71, Osaka, Japan). For the quantitative analysis of ARS staining after 14 days. The calcium concentration in the deposited minerals was quantified by adding 10% acetic acid (Sigma Aldrich, St. Louis, MO, USA). The absorbance of the dissolved calcium nodules was measured via microplate reader at 415 nm.

#### 2.7.4. Angiogenesis Related Detection

A Transwell assay was performed to evaluate cell migration. Briefly, HUVECs were inoculated upside of the Transwell (12-well plates, Corning, Union City, CA, USA) with an 8 μm pore size at a concentration of 1 × 10^4^ cells/well, and leaching liquids (350 μL/well) were added in the lower chamber. After 24 h, the cells in the upper chamber were removed using a swab rod and crystal violet was used to image the cells that had migrated to the bottom. An inverted microscope (Olympus, IX71, Osaka, Japan) was used for observation, and quantitative analysis was performed using ImageJ software (NIH, Bethesda, MD, USA).

In addition, HUVECs were introduced as an in vitro model to evaluate angiogenesis. Growth factor reduced Matrigel (BD Biosciences, San Jose, CA, USA) was mixed with leaching liquids (2:1, *v*/*v*) in 24-well plates and polymerized for 30 min. HUVECs (12-well plates, 1 × 10^4^ cells/well) were then inoculated onto the Matrigel surface and incubated with leaching liquids (350 μL/well) for 6 h. Microscopic images were captured at random using an inverted microscope (Olympus IX71, Japan) to quantify the total segment length using the Angiogenesis Analyzer in ImageJ (NIH, Bethesda, MD, USA).

Immunofluorescence staining was used to further evaluate angiogenesis in vitro. The experimental procedures were performed as previously reported [16]. Briefly, HUVECs were cultured in 6-well plates with leaching liquids at 4 × 10^4^ cells/well. After incubating after 3 days, collected HUVECs were incubated with the primary antibodies overnight at 4 °C, followed by incubating with second antibodies for 1 h at room temperature (RT). After rinsing with PBS, the collected HUVECs were stained with DAPI for 5 min and observed. Quantification analysis was obtained by LAS X software 1.4.5. The antibodies used in this study are listed in Supplementary Table S1.

#### 2.7.5. Expression Levels of Osteogenesis and Angiogenesis-Related Genes

The cells were cultured in 6-well plates with leaching liquids at 4 × 10^4^ cells/well. After 7 days of co-culture, total RNA was extracted using a 1 mL/well TRIzol RNA Extraction Kit (Invitrogen, Carlsbad, CA, USA). Next, the RNA was reverse transcribed into complementary DNA using the Revert Aid First Strand Complementary DNA Synthesis Kit (Thermo Fisher Scientific, San Jose, CA, USA). Quantitative reverse transcription-polymerase chain reaction (qRT-PCR) was used to examine the gene expression levels of OCN, OPN, Col-1, Runx-2, VEGF, and HIF-1α; human β-actin was set as a control. The sequences are designed from NCBI and synthesized by Servicebio, which are listed in Supplementary Table S1.

#### 2.7.6. Expression Levels of Osteogenesis and Angiogenesis-Related Proteins

Western blotting was used to evaluate the expression levels of osteogenesis- and angiogenesis-related proteins. The cells were cultured with leaching liquid in a 6-well plate (8 × 10^4^ cells/well) for 7 days. The leaching liquid was changed every 2 days. Finally, the treated cells were lysed and collected, the total protein was calculated by BCA assay kit. Next, the total protein with a volume of 20 μg was separated by 10% SDS-PAGE gels, and further electro-transferred to the PVDF membrane. After rinsing with TBST and blocking those non-specific adsorption sites using a 5% nonfat dry milk solution for 2 h, the above bands were incubated with specific primary antibodies (anti-OCN, anti-OPN, anti-COL-1, anti-Runx2, anti-VEGF and anti-HIF-1α) overnight at 4 °C. Afterwards, the antibody-bound samples were then incubated with corresponding secondary antibodies for 1 h. The protein level was normalized to β-actin. Lastly, the results were obtained via a ChemiDoc Imaging System (Bio-Rad, Hercules, CA, USA) and the grayscale of the protein bands was quantified using ImageJ (NIH, Bethesda, MD, USA). The used antibodies and relevant parameters are presented in Table S2.

### 2.8. In Vivo Evaluation of the Scaffolds

#### 2.8.1. Establishment of Animal Model

The surgical procedure was approved by the Animal Committee of the Tongren Hospital, Wuhan University (Wuhan, China). (Ethics approval number: SY2022-021). Briefly, thirty Sprague Dawley rats (male, 2 months old, 300 ± 30 g) were randomly divided into three groups as follows: (1) the control group (blank control, *n* = 10), (2) the MPC group (negative control, *n* = 10), and (3) the OMPC group (experimental group, *n* = 10). All animals received general anesthesia with 4% isoflurane and maintained with 2% isoflurane. The surgical area was shaved and disinfected using 75% ethanol. A 2 cm incision was made to reveal the skull, and a surgical drill was used to create bilateral cranial defects (d = 6 mm, h = 2 mm). The sterilized scaffolds (MPC and CMPC, d = 6 mm, h = 2 mm) were implanted into the defect site, and the layers of subcutaneous tissue were closed using absorbable sutures.

Excessive amounts of isoflurane gas were administered at four- and eight-weeks post-implantation. Five rats were sacrificed at each designated time point and the harvested skulls were fixed in 10% formalin solution (Servicebio, Wuhan, China) for subsequent experiments.

#### 2.8.2. Micro-CT Analysis

Following fixing in 10% formalin solution for 24 h, the reconstructions of the harvested skulls were evaluated by Micro-CT (Bruker SkyScan1172, Belgium, voltage 100 KV, current 100 μA, pitch 2, filter 0.11 mm Cu). Postoperative bone formation was assessed by measuring the ratio of new bone volume to tissue volume (BV/TV) and bone mineral density (BMD).

#### 2.8.3. Histological Analysis

The harvested skulls were fixed and rehydrated in ethanol. After dehydration, the samples were embedded in paraffin. Following the embedding of methyl (Biotechnology, Wuhan, China) and till solidification, 300 μm thick sections were allotted for hard tissue staining. Hematoxylin and eosin (H&E) and Masson staining were performed for histological analysis. Immunohistochemical staining was performed on the harvested subcutaneous samples using standard protocols on paraffin sections stained with primary antibodies against CD31 and OPN (1:800 and 1:600, Beyotime, Wuhan, China), followed by counterstaining with hematoxylin, and the number of positively stained cells in the specimen was calculated. ImageJ was used for quantitative analysis.

#### 2.8.4. Systematic Biocompatibility

The harvested viscera, including heart, liver, spleen, lungs, and kidneys, were kept in 10% formalin for 24 h after eight weeks and then dehydrated using different grades of ethanol. Sections of 50 μm thickness were allotted for tissue staining after embedding of methyl methacrylate (Biotechnology, Wuhan, China) until solidification. Hematoxylin and eosin staining was performed for histological analysis.

### 2.9. Statistical Analysis

Data were illustrated as mean ± SD and statistical analyses were employed using Origin 2023b software. One-way analysis of variance (ANOVA), followed by Tukey’s post hoc test, was used to determine statistical significance. Differences were considered significant at *p* < 0.05.

## 3. Results and Discussion

### 3.1. Preparation and Characterization of Fabricated Scaffolds

The surface of the bioceramics, which is the primary step for their interaction with the surrounding bone tissue, can play a critical role in their biological fate [17]. The traditional strategy is to form a bone-like layer which can form chemical bonds with the surrounding bone tissue while improving mechanical properties for osseointegration [18,19]. However, the formation of an apatite layer represents a seesaw effect between biodegradation and surface bioactivity. Qin et al. fabricated wollastonite-hydroxyapatite (WS-HA) coated bio-nanocomposite by electrophoretic deposition (EPD), and the apatite-like layer led to improved biocompatibility and mechanical properties while reducing the degradation rate [20].

**Figure 1:**
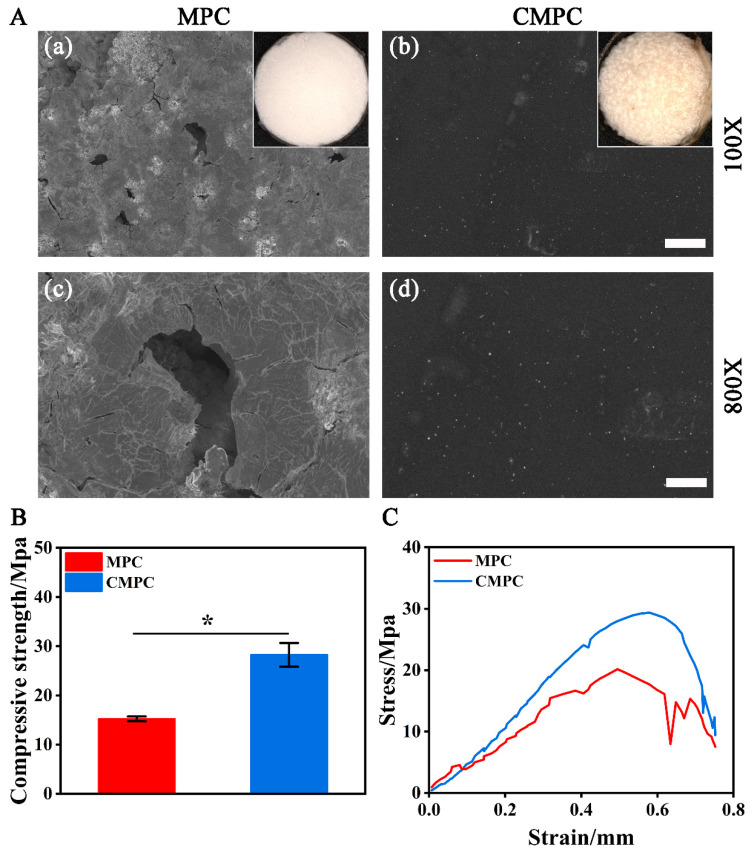
(**A**) Optical and SEM images of MPC (**a**,**b**) and CMPC (**c**,**d**). (**B**) Compressive strength. (**C**) Representative compression stress–strain curves. Error bars represent means ± SD for *n* = 3. Scale bar: (**A**) 100 μm for (**a**,**b**), 10 μm for (**c**,**d**). Differences were identified as significant with *, represented by *p* < 0.05.

In this study, by lyophilizing CS solution onto k-struvite, the CMPC was successfully prepared with a smooth surface (Figure 1A) and significantly enhanced compressive strength (Figure 1B,C, 15.23 ± 0.48 Mpa for MPC and 28.26 ± 2.41 Mpa for CMPC, *p* < 0.05). Interestingly, although the mechanical properties of the CS coating reported in the literature is much lower than that of MPC [21], the compressive strength of the CMPC is approximately 1.85 times higher than MPC. We speculated that the negative charge of N-acetylgalactosamine forms an ion adsorption with the cations of MPC, which eventually leads to a filling effect into the pores.

**Figure 2:**
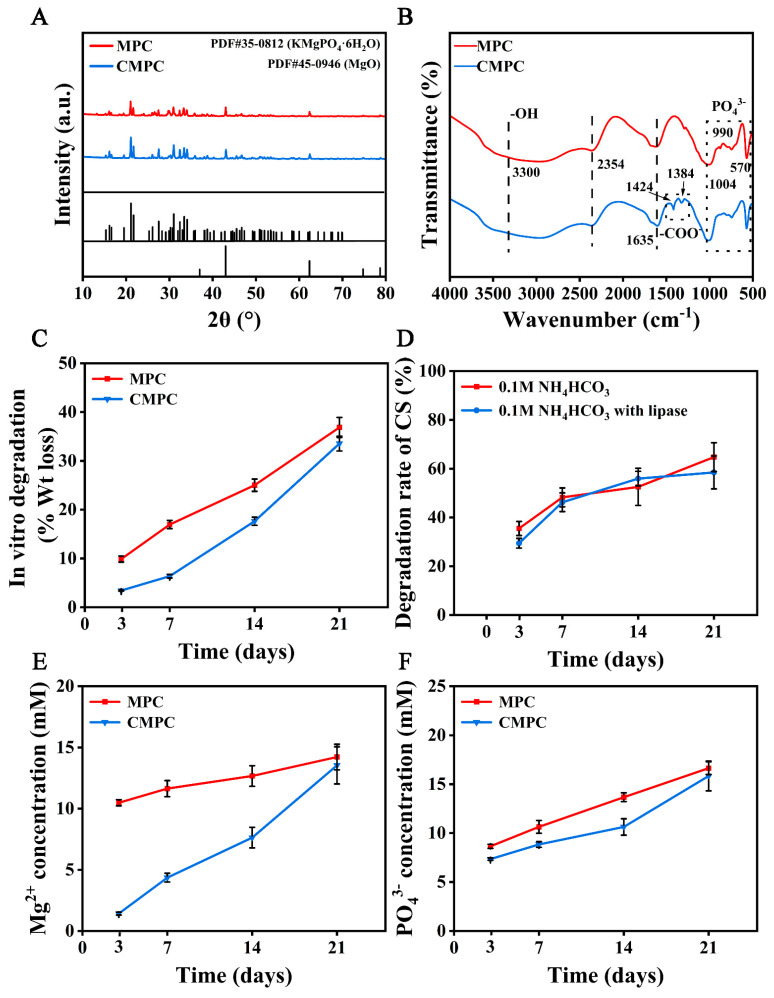
(**A**) X-ray diffraction patterns. (**B**) FTIR spectrum. (**C**) In vitro degradation rate of scaffolds. (**D**) In vitro degradation rate of CS coating. (**E**) Release concentration of Mg^2+^. (**F**) Release concentration of PO_4_^3−^. Error bars represent means ± SD for *n* = 3.

Different from the formation of an apatite layer, which requires the catalytic effect of the Si–OH groups and Ti–OH groups for nucleation [22], the lyophilization method preserves the intact structure of the functional groups by mixing the polymer solution with the substrate materials. As shown in Figure 2A, the XRD pattern confirmed the representative characteristic peaks of KMgPO_4_·6H_2_O crystals and residual MgO with complete depletion of KH_2_PO_4_, which matched with the identification card (PDF 35-0812 and 45-1946) reported formerly. In addition, the intensive peaks of PO_4_^3−^ (570 and 1004 cm^−1^) and OH^−^ (3300 cm^−1^) vibration, indicative of KMgPO_4_·6H_2_O, were observed both in the MPC and CMPC scaffolds, while the characteristic peaks for C-O and C=O stretching vibration at 1384 and 1424 cm^−1^ proved the existence of the glucuronic acid form of CS (Figure 2B). The above results indicate that the lyophilization method is a simple and controllable preparation technique for improving surface bioactivity.

CS can inhibit cartilage degeneration by regulating enzyme-related degradation [15,23]. But how CS regulates biodegradation-related bone regeneration is still unclear. Biodegradation is mainly divided into two parts: physical dissolution and enzymatic degradation [24]. As illustrated in Figure 2C, the degradation rate of the MPC group (36.85 ± 2.05%) was higher than that of the CMPC group (33.54 ± 1.52%) at day 21, suggesting the prevention of the crystal dissolution. For the enzymatically involved part, we evaluated the degradation of the CS coating in the lipase-containing liquid. As shown in Figure 2D, the addition of lipase did not significantly improve the degradability of the CS coating, indicating that the β-(1–4) glycosidic bonds are structurally intact and stable in the enzymatic degradation environment.

Controlled release of Mg^2+^ during biodegradation remains a challenge in MPCs. The commonly used method is to change the pore size or topological structure [25,26]. In this study, we utilized the ion adsorption ability of the CS coating to achieve this goal. Consistent with the trend in degradation rate, the Mg^2+^ concentration of the MPC group was higher than that of the CMPC group, and the highest concentration reached 15.64 ± 1.05 mM at day 21. Conversely, the release rate of PO_4_^3−^ remained relatively constant (Figure 2C), indicating that the CS coating had a low affinity for anions, which has been verified in other studies [27,28].

### 3.2. Effect of CS Coating on In Vitro Biocompatibility

**Figure 3:**
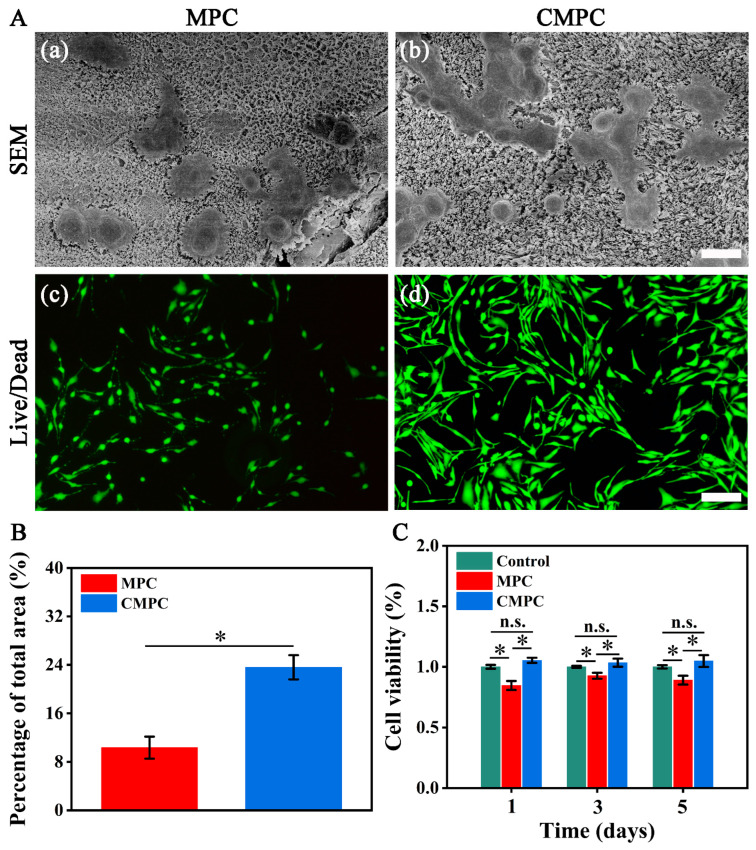
(**A**) SEM images (**a**,**b**) and fluorescence images of live/dead staining (**c**,**d**) of seeded cell on MPC and CMPC. (**B**) Quantitative analysis of the adhesion area. (**C**) CCK-8 assay. Error bars represent means ± SD for *n* = 3. Scale bar: (**A**) 100 μm for (**a**–**d**). Differences were identified as significant with *, represented by *p* < 0.05. n.s. means no significant difference.

Good cytocompatibility is a critical prerequisite for the use of magnesium implants for bone regeneration. The adhesion of cells to scaffolds is essential for osteoblast survival and matrix mineralization [29,30]. In this study, the cell morphology on the scaffold surface is depicted in Figure 3(Aa,Ab). More cells were connected to sheets with extended pseudopodia in the CMPC group. A live/dead assay was used to evaluate cell viability. As illustrated in Figure 3(Ab,Ac), all groups presented fluorescence of MC3T3-E1s in green without red, suggesting the good cytocompatibility of MPC and CMPC. However, as shown in Figure 3B, the adhesion area of the CMPC group was significantly higher than that of the MPC group (*p* < 0.05), suggesting that the CS coating facilitated cell adhesion in the early stage. Cell proliferation was evaluated using a CCK-8 assay. When cultured with the leaching liquids, the CMPC and control groups displayed better cell viability than the MPC group, while the CMPC group possessed the highest. It has been reported that the cell adhesion behavior on the surface of bioceramics is affected by numerous factors, among which the released ion concentration and surface bioactivity are critical [31,32]. For Mg^2+^, concentrations between 2–10 mM appear to represent an optimal range for exerting physiological effects [33,34]. Combined with the results presented in Figure 2D, the CS coating exerted synergistic effects via its functional groups and cation adsorption ability to mediate the controlled release of Mg^2+^, leading to differences in the cell adhesion area between the MPC and CMPC groups.

### 3.3. Effect of CS Coating on In Vitro Osteogenesis

**Figure 4:**
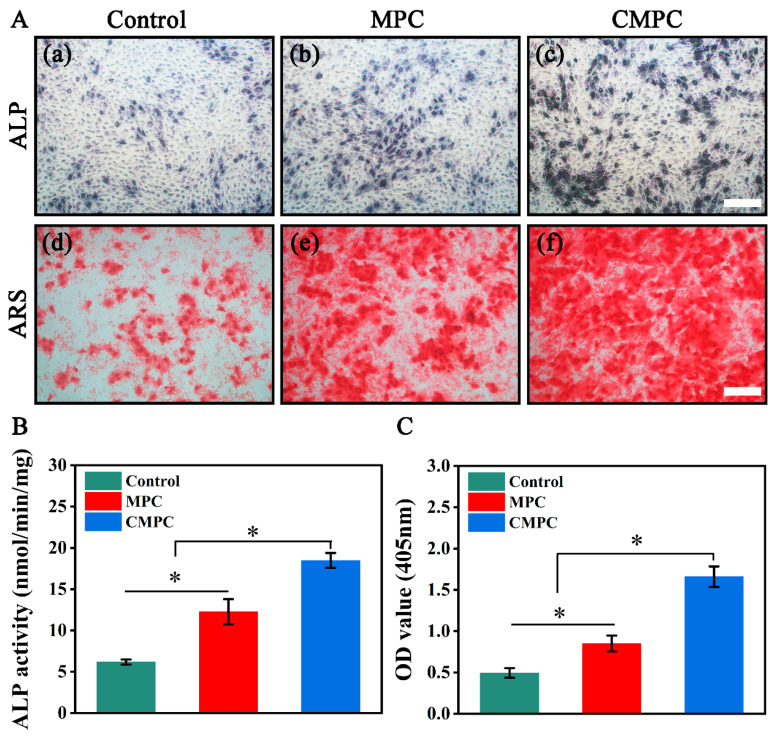
(**A**) ALP staining (**a**–**c**) and ARS staining (**d**–**f**) of MC3T3-E1s. (**B**) ALP activity of MC3T3-E1s. (**C**) Quantitative analysis of ARS staining. Error bars represent means ± SD for *n* = 3. Scale bar: (**A**) 100 μm for (**a**–**f**). Differences were identified as significant with *, represented by *p* < 0.05.

Ideal bone substitutes must have favorable in vitro osteogenic activity before implantation. ALP activity is one of the most widely used biomarkers of early osteoblastic differentiation [35,36]. In the present study, ALP-related assays were performed to assess the early osteogenic ability of MC3T3-E1s cultured in the leaching liquids. As depicted in Figure 4(Aa–Ac), the number of ALP nodules in the CMPC group was higher than that in the MPC and control groups on day 7. This trend was verified by the quantitative analysis of ALP activity, and the CMPC group displayed the highest OD values (Figure 4B). Biomineralization is another critical index for evaluating osteogenesis in later stages [37]. After co-culturing for 21 days, calcium deposition-related assays were performed. Consistent with the results of the ALP-related assay, the CMPC group expressed a more mineralized matrix and showed a significantly higher level of calcium deposition than the MPC and control groups (Figure 4(Ad–Af),C), indicating that the CMPC group possesses osteogenic advantages. These results indicate that the immobilization of the CS coating was more conducive to inducing early and later osteogenic differentiation.

**Figure 5:**
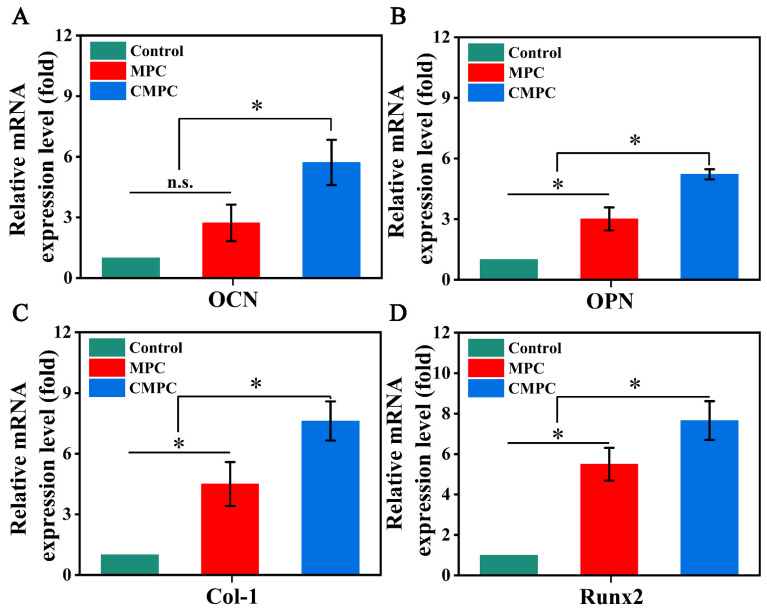
Effect of CS coating on the expression of osteogenesis-related genes encoding ocn (**A**), opn (**B**), col-1 (**C**), and runx2 (**D**). Error bars represent means ± SD for *n* = 3. Differences were identified as significant with *, represented by *p* < 0.05. n.s. means no significant difference.

The expression levels of osteogenesis-related genes and proteins are shown in Figure 5 and Figure S1. The gene and protein expression level of ALP, OCN, COL 1, and Runx2 in the CMPC group was higher than that in the MPC and control groups on day 7. Recently, the effects of Mg^2+^ supplementation on osteogenesis have become increasingly intricate [38]. Zheng et al. demonstrated that supplementation with Mg^2+^ (10 mM) at an early stage promotes osteoblast differentiation while inhibiting subsequent mineralization [1]. In this study, via combining the results in Figure 2, we proved that the controlled release of Mg^2+^ (<8 mM) at an early stage might be beneficial for late mineralization.

### 3.4. Effect of CS Coating on In Vitro Angiogenesis

**Figure 6:**
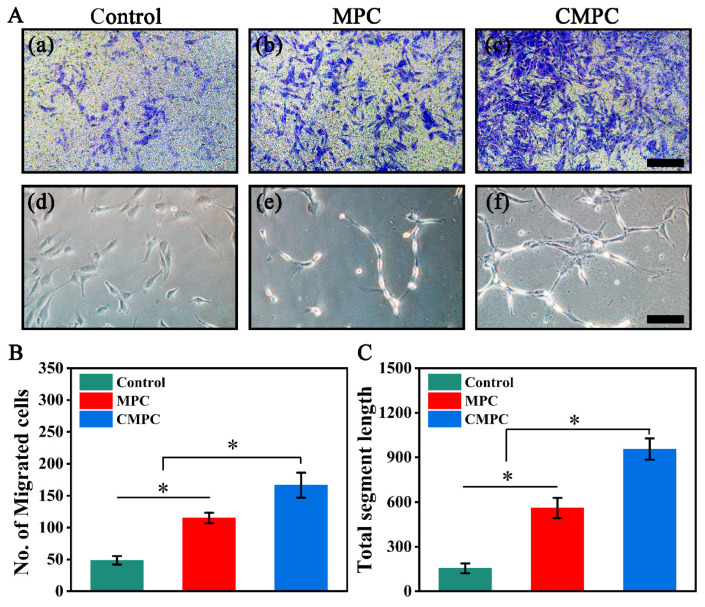
Effect of CS coating on the cell migration (**A**) (**a**–**c**) and tube formation (**d**–**f**) of HUVECs. (**B**) Quantitative analysis of the Transwell assay. (**C**) Quantitative analysis of the tube formation assay. Error bars represent means ± SD for *n* = 3. Scale bar: (**A**) 100 μm for (**a**–**f**). Differences were identified as significant with *, represented by *p* < 0.05.

Vascularization is a critical physiological process coupled with osteogenesis during bone formation, and the ability of magnesium implants to regulate angiogenesis requires further investigation [39]. In this study, Transwell and tube formation assays were used to investigate the effect of the CS coating on angiogenesis. As illustrated in Figure 6(Aa–Ac), more stained HUVECs were observed in the CMPC group than in the other groups, which was verified by quantitative analysis (Figure 6B). Moreover, as depicted in Figure 6(Ad–Af), the CMPC group exhibited the highest tube formation ability, as expected, and more stripes were obtained, as confirmed by quantitative analysis (Figure 6C).

**Figure 7:**
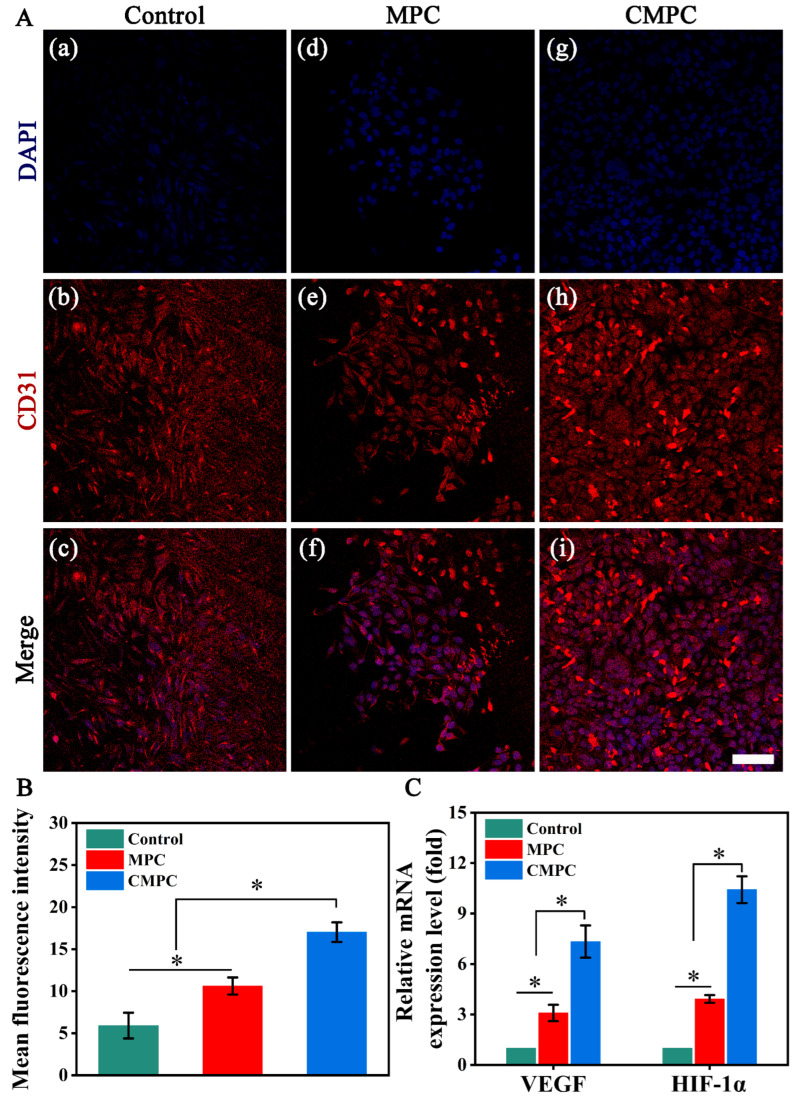
(**A**) Immunofluorescence images stained with CD31 (red) and DAPI (blue), and (**a**–**c**) for Control, (**d**–**f**) for MPC, (**g**–**i**) for CMPC. (**B**) Quantitative analysis of the fluorescence intensity. (**C**) qPCR analysis to assess the expression of angiogenesis-related genes (VEGF and HIF-1α). Error bars represent means ± SD for *n* = 3. Scale bar: (**A**) 200 μm for (**a**–**i**). Differences were identified as significant with *, represented by *p* < 0.05.

As specific indicators during angiogenesis, the relative expression levels of CD31, VEGF, and HIF-1α were, respectively, analyzed by immunofluorescence, qPCR and Western blotting. Compared with the MPC and control groups, HUVECs cultured in the CMPC group displayed higher expression level of CD31 (Figure 7A), as verified by quantitative analysis (Figure 7B). The aforementioned results were verified through qPCR and Western blotting (Figure 7C and Figure S2), where CMPC group exhibited the highest expression level of VEGF and HIF-α, suggesting the CS coating improved the angiogenic capacity. Interestingly, the angiogenesis-related indices of the MPC group remained higher than those of the control group, which might be related to the fact that the optimal dosage of Mg^2+^ for promoting osteogenesis inevitably varies according to the cell line. Similar results have also been reported in other studies [40,41].

Previous studies revealed that Mg^2+^ is involved in the induction of angiogenesis, while preparing a suitable delivery system to control ion concentration remains a challenge. Lin et al. prepared a Mg-enriched 3D culture system to promote vascularized bone regeneration [42]. However, Mg ions were used as an additive to the gel system, which was difficult to achieve sustained release. In this study, as a magnesium source, MPC can provide Mg^2+^ continuously until complete biodegradation, and the concentration is controlled through the CS coating, which is beneficial to long-term bone regeneration. Moreover, HIF-1α is a transcription factor that promotes angiogenesis by simulating hypoxic conditions and promoting the transcription of VEGF [43,44]. In this study, the increased expression of HIF-1α results in enhanced VEGF, which is consistent with the previous studies on magnesium implants [45].

### 3.5. Effect of CS Coating on In Vivo Bone Regeneration

**Figure 8:**
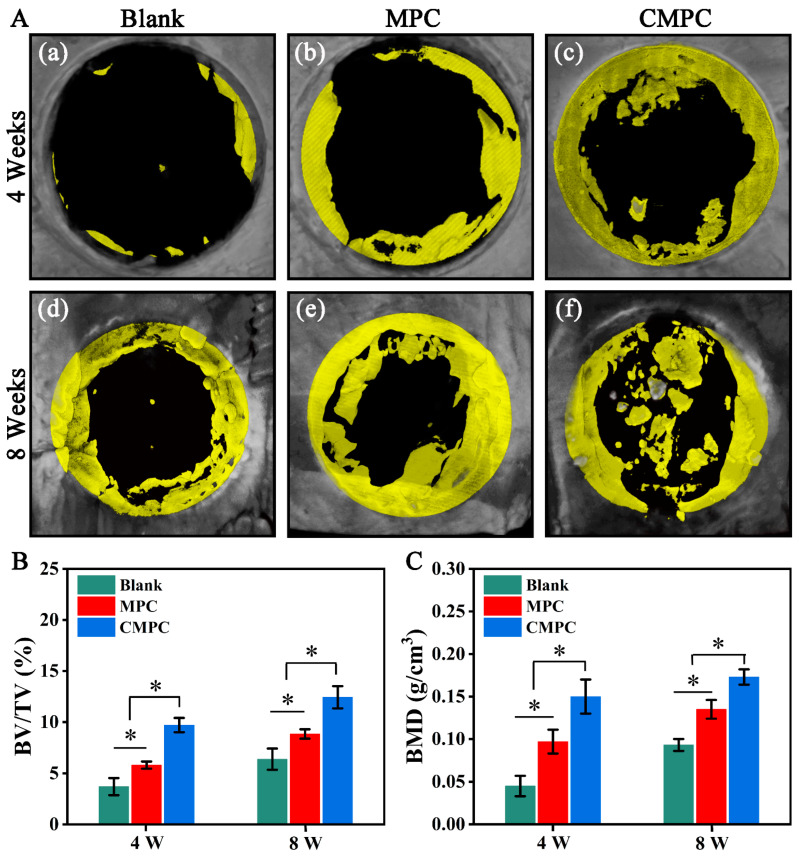
(**A**) Micro-CT reconstruction of calvarial defects after 4 and 8 weeks, and (**a**,**d**) for Blank, (**b**,**e**) for MPC, (**c**,**f**) for CMPC. (**B**) Quantitative analysis of BV/TV. (**C**) Quantitative analysis of BMD. Error bars represent means ± SD for *n* = 5. Differences were identified as significant with *, represented by *p* < 0.05.

As a supplement for in vitro assessments of osteogenesis and angiogenesis, the cranial defect model was widely accepted to evaluate bone regeneration ability in vivo [46,47]. As illustrated in Figure 8A, the ROI in the control group demonstrated minimal mineralized new bone ingrowth after 4 weeks. New bone ingrowth was observed in MPC and CMPC groups. In addition, new bone ingrowth was visibly enhanced in the CMPC group compared to the MPC group. Specifically, the center of the defect regions in the CMPC group was largely occupied by bony tissue ingrowth. For quantitative analysis (Figure 8B,C), the values of BV/TV and BMD were higher in the MPC and CMPC groups than in the control group after 4 and 8 weeks, respectively, demonstrating enhanced bone repair in the scaffold implantation groups. Moreover, compared to the MPC group, the BV/TV and BMD values were distinctly higher in the CMPC group, indicating better bioperformance for stimulating bone regeneration in the CMPC group.

**Figure 9:**
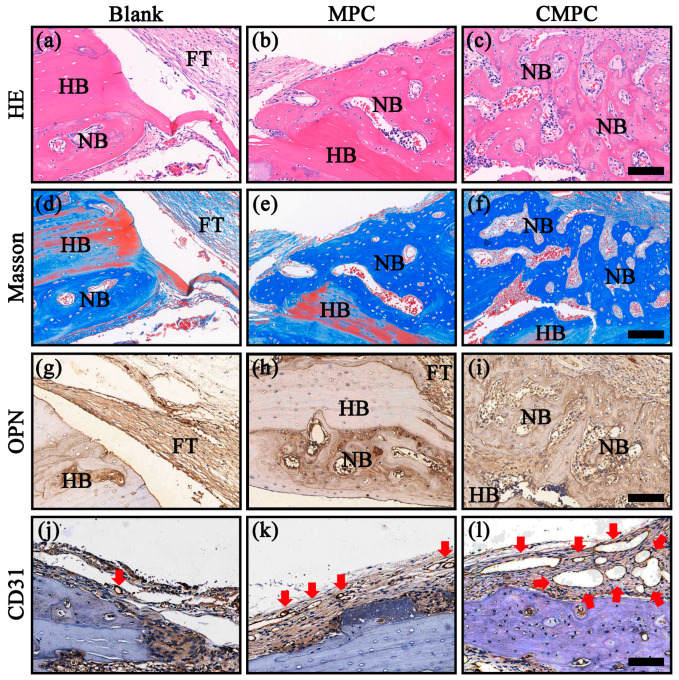
H&E staining (**a**–**c**), Masson’s trichrome staining (**d**–**f**), and immunohistochemical staining of OPN (**g**–**i**), CD31 (**j**–**l**) after 8 weeks. Scale bar: 200 μm for (**a**–**l**). HB: host bone; NB: new bone; FT: fiber tissue; red arrows: blood vessels.

As illustrated in Figure 9a–c, fiber tissues occupied by a few marginal bone tissues were observed in the HE staining of the control group, consistent with the outcome of micro-CT analysis. In comparison, certain amounts of new bone ingrowth were observed in both the MPC and CMPC groups, particularly in the CMPC group, which induced a trabecular-like new bone structure. This phenomenon was validated by Masson’s trichrome staining (Figure 9d–f); the number of blue-stained collagen fibers in the CMPC group was higher than that in other groups. Furthermore, as biomarkers of in vivo osteogenesis and angiogenesis, OPN and CD31 were evaluated. As shown in Figure 9g–l and Figure S3, more positive areas of immunohistochemical staining were detected in the CMPC group than in the other groups (*p* < 0.05). Combined with HE and Masson staining, these results demonstrate that CS coating endows MPC with enhanced osteogenesis and angiogenesis in vivo.

**Figure 10:**
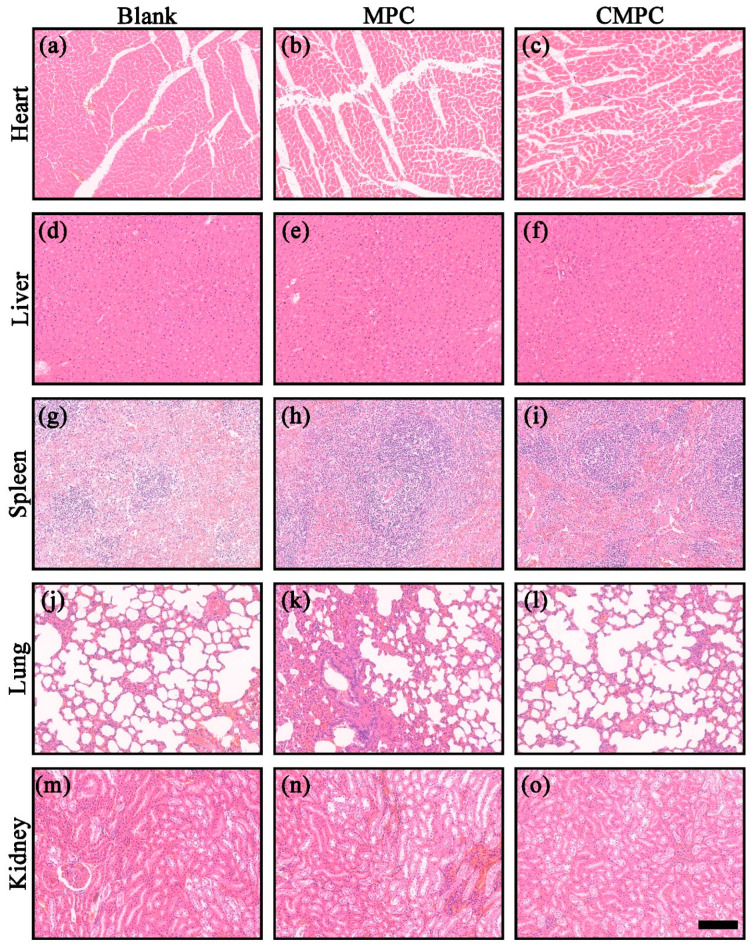
H&E staining of major organs (heart (**a**–**c**), liver (**d**–**f**), spleen (**g**–**i**), lung (**j**–**l**), and kidney (**m**–**o**)) after 8 weeks. Scale bar: 200 μm for (**a**–**o**).

Meanwhile, the systematic biosafety evaluation in Figure 10a–o and Figure S4 demonstrated that the histological morphology of the major organs (heart, liver, spleen, lung, and kidney) and blood index (AST and Mg^2+^) were normal, and there was no significant difference among all groups. In particular, as a major pathway involved in Mg^2+^ metabolism, a common glomerulus structure could be found, further indicating the biological safety and biocompatibility of the MPC and CMPC scaffolds.

According to recent studies, CS improved the affinity with surrounding tissues in vivo experiments by mimicking extracellular matrix components [48]. In this work, we further demonstrate that it can also control the ion release rate of magnesium-based bone cement through cation adsorption capacity, finally leading to a Mg^2+^ concentration-dependent osteogenesis and angiogenesis.

### 3.6. Limitations of This Study

Indeed, the correlation between the in vitro and in vivo optimized concentrations of Mg^2+^ remains controversial [49,50]. The lack of in vitro models that accurately replicate in vivo microenvironments poses a significant obstacle, and new functional biomimetic models should be designed and adopted in future studies.

## 4. Conclusions

In this study, we report a lyophilization strategy for coating the surface of k-struvite with CS solution. The physicochemical properties, in vitro osteogenesis and angiogenic ability of CMPC were significantly enhanced without phase change in the substrate. In addition, the ideal in vitro/vivo biocompatibility was achieved. Importantly, the results indicated that CS increased the cell adhesion area through its functional groups, and induced the desired biodegradation rate of the CMPC, finally mediating controlled release of Mg^2+^ via its cation-adsorption capacity. Moreover, concentration-dependent osteogenesis and angiogenesis in vivo were observed, indicating the excellent regenerative bioperformance of the CMPC in bone regeneration. We believe that this easy and efficient strategy will inspire the development of novel polymer-coated bioceramics and provide new choice for the treatment of bone defects in the future. 

## Data Availability

Data are contained within the article and Supplementary Materials.

## Supplementary Materials

The following supporting information can be downloaded at: https://www.mdpi.com/article/10.3390/biomedicines12010074/s1, Figure S1. Effect of CS coating on the expression of osteogenesis related proteins containing (A) OCN, OPN, COL-1 and Runx2. (B–E) Relative bands intensity of OCN, OPN, COL-1 and Runx2; Figure S2. Effect of CS coating on the expression of angiogenesis related proteins containing (A) VEGF and HIF-1α. (B,C) Relative bands intensity of VEGF and HIF-1α; Figure S3. Effect of CS coating on the expression of OPN (A) and CD31 (B) in immunohistochemistry; Figure S4. Effect of CS coating on the expression of AST (A) and Mg^2+^ (B) in blood serum from SD rats; Table S1. Primer sequences performed in qPCR assay; Table S2. Antibodies used in this study.
