# Supplementary material for: Functionalized Magnesium Phosphate Cement Induces In Situ Vascularized Bone Regeneration via Surface Lyophilization of Chondroitin Sulfate
Source: Biomedicines. 2023 Dec 28;12(1):74. doi: 10.3390/biomedicines12010074 (PMC10812989; doi:10.3390/biomedicines12010074)
Supplement: Supplementary file 1 [file biomedicines-12-00074-s001.zip › biomedicines-2660606-supplementary.pdf]

Supplementary Information

Functionalized magnesium phosphate cement induces in situ vascularized bone regeneration via surface lyophilization of chondroitin sulfate.

Changtian Gong[1,2], Jian Yang[1], Xiping Zhang[1], Zhun Wei[1], Xingyu Wang[1], Xinghan Huang[1], Ling Yu[1*] and Weichun Guo[1*]

[1] Department of Orthopedics, Renmin Hospital of Wuhan University, Wuhan 430060, China
[2] Center of Regenerative Medicine, Renmin Hospital of Wuhan University, Wuhan 430060, China
* Correspondence: **Correspondence to:** Prof. Weichun Guo, Renmin Hospital of Wuhan University, Wuhan, 430060, China, Email: guoweichun@aliyun.com

Supplementary figures

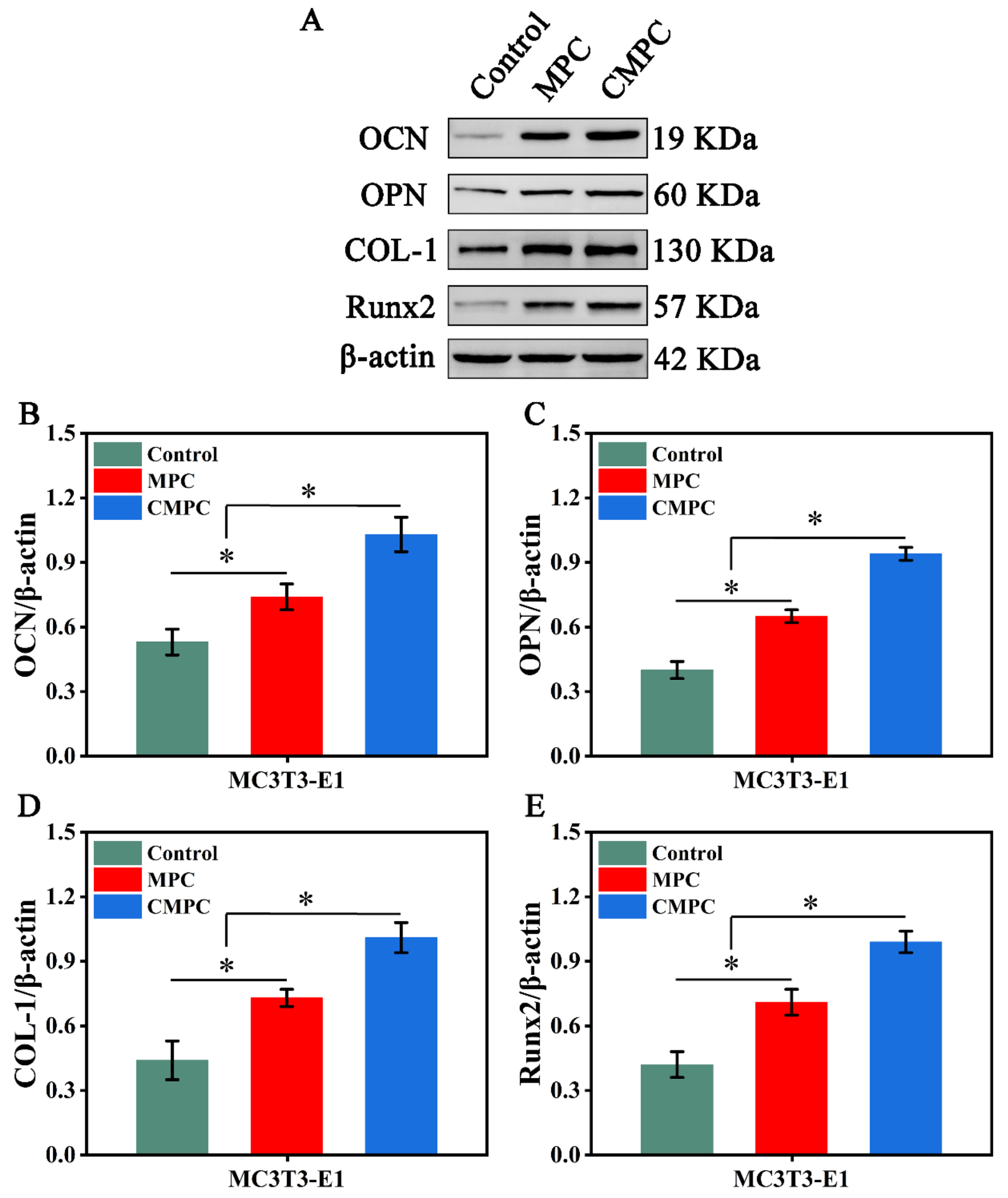

Figure S1. Effect of CS coating on the expression of osteogenesis related proteins containing (A) OCN, OPN, COL-1 and Runx2. (B-E) Relative bands intensity of OCN, OPN, COL-1 and Runx2. Error bars represent means ± SD for n = 3. Differences were identified as significant with *, represented by a $P < 0.05$.

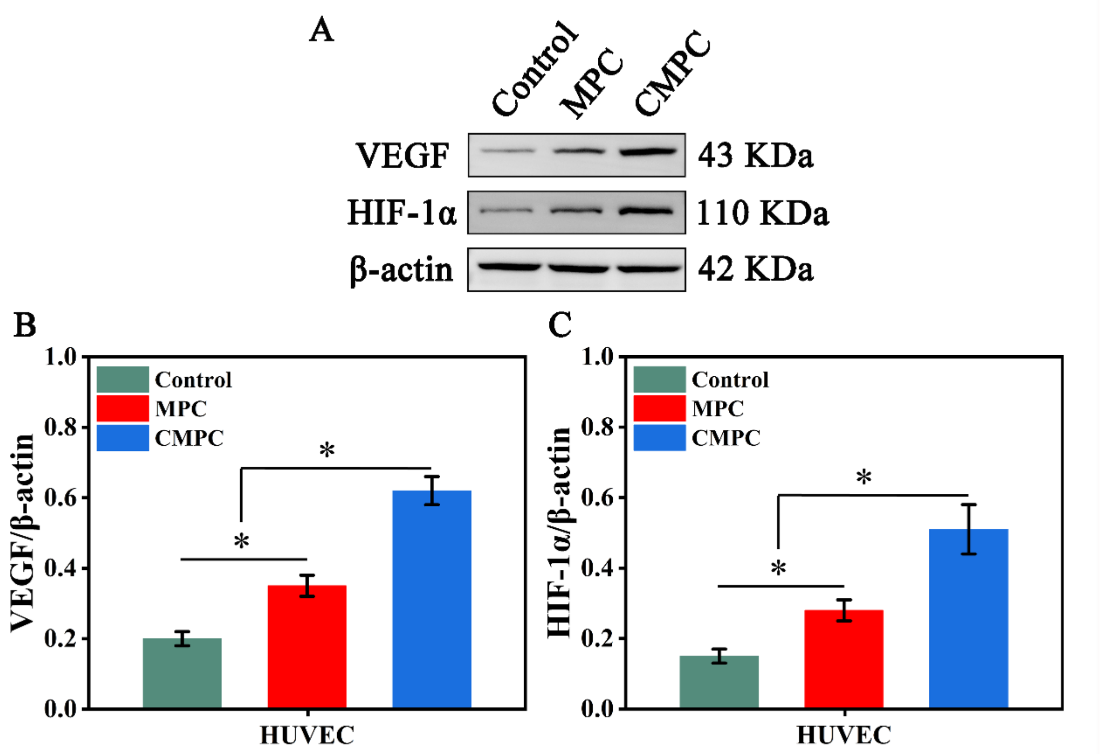

Figure S2. Effect of CS coating on the expression of angiogenesis related proteins containing (A) VEGF and HIF-1α. (B-C) Relative bands intensity of VEGF and HIF-1α. Error bars represent means ± SD for n = 3. Differences were identified as significant with *, represented by a $P < 0.05$.

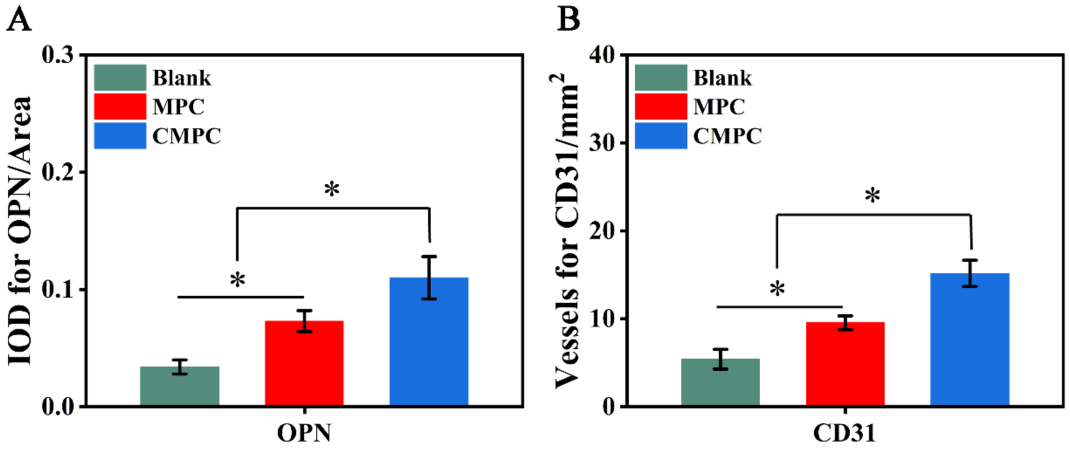

Figure S3. Effect of CS coating on the expression of OPN (A) and CD31 (B) in immunohistochemistry. Error bars represent means ± SD for n = 3. Differences were identified as significant with *, represented by a $P < 0.05$.

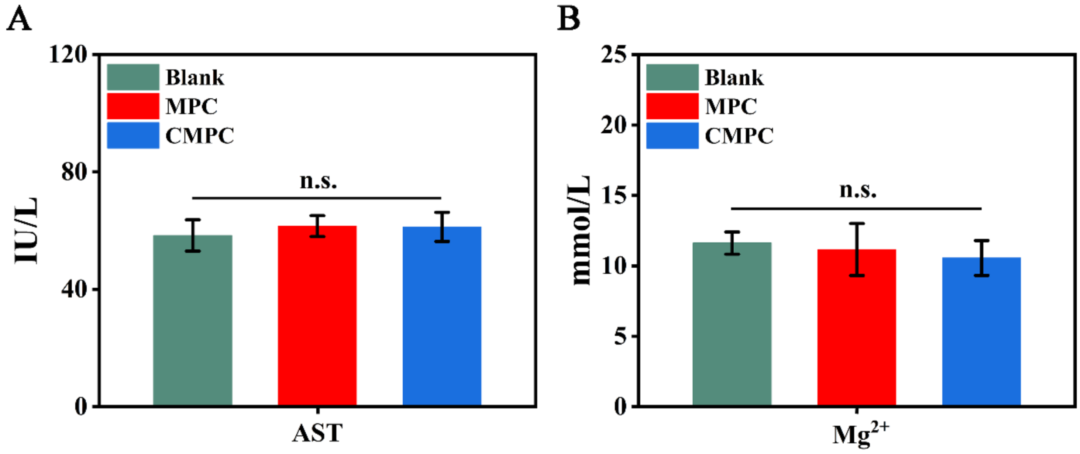

Figure S4. Effect of CS coating on the expression of AST (A) and Mg^{2+} (B) in blood serum from SD rats. Error bars represent means ± SD for n = 3. Differences were identified as significant with *, represented by a $P < 0.05$.

Supplementary tables

Table S1. Primer sequences performed in qPCR assay.

Gene	Forward primer	Reverse primer
**OCN**	F: CGCCTACAAGCGCATCTATG	R: AGCTGTGCCGTCCATACTTTC
**OPN**	F: GATAGTGTTTTGGGCCCTGAG	R: TCCTGTAAGTTTGCCTGCCTC
**Col-1**	F: CTCCCCAGTTGTGATCTGGC	R: GGACCCAGAGTCCACCTCTCT
**Runx2**	F: TGGGTATAAGTCCCTTTCTGCC	R: CTGGTGGGACGATTCAGCAT
**β-actin**	F: GCTGTGCTATGTTGCCCTAGAC	R: CCGCTCATTGCCGATAGTGATG
**VEGF**	F: CTTGCCTTGCTGCTCTACCT	R: GCAGTAGCTGCGCTGATAGA
**HIF-1α**	F: TGTACCCTAACTAGCCGAGGA	R: CTGTGCAGTGCAATACCTTCC
**β-actin**	F: GCACTCTTCCAGCCTTCCTT	R: AATGCCAGGGTACATGGTGG

Table S2. Antibodies used in this study

Antibodies	Application	Source	Catalogue number
anti-CD31	IF	Servicebio	GB11063-2
Alexa Fluor® 647	IF	Abcam	ab150079
anti-OPN	WB	ABclonal	A19092
anti-OCN	WB	ABclonal	A6205
anti-COL-1	WB	ABclonal	A1352
anti-Runx2	WB	ABclonal	A2581
anti-VEGF	WB	ABclonal	A5708
anti-HIF-1α	WB	ABclonal	A24002
anti-β-actin	WB	CST	8457
